# An Easy-to-Use Risk Stratification System for NSTE-ACS Patients Combining Autonomic Nervous System and Coronary Physiology

**DOI:** 10.7150/ijms.111214

**Published:** 2025-04-28

**Authors:** Xiaomeng Yang, Zeyan Li, Xinyu Liu, Tianyou Xu, Fu Yu, Shoupeng Duan, Qiang Deng, Lang Wang, Zhuo Wang, Hong Jiang, Lilei Yu

**Affiliations:** Cardiovascular Hospital, Renmin Hospital of Wuhan University; Hubei Key Laboratory of Autonomic Nervous System Modulation; Cardiac Autonomic Nervous System Research Center of Wuhan University, Wuhan, Hubei, 430060, P.R. China.

**Keywords:** non-st-elevation ACS, autonomic nervous system, quantitative flow ratio, major adverse cardiac events, risk stratification system

## Abstract

**Background:** The evaluation of autonomic nervous system (ANS) function and coronary physiology through quantitative flow ratio (QFR) analysis provides a precise method for assessing the severity and prognosis of acute coronary syndrome (ACS).

**Aims:** This study aimed to develop and validate a risk score model for predicting the long-term prognosis of non-ST-elevation ACS (NSTE-ACS) patients who underwent complete and successful percutaneous coronary intervention (PCI).

**Methods:** NSTE-ACS patients who underwent complete and successful PCI with preoperative and postoperative QFR measurements between January 2018 and December 2020 in our medical center were included. 24-hour Holter monitoring was performed to assess deceleration capacity (DC) and heart rate variability (HRV) parameters. The primary endpoint was the occurrence of major adverse cardiac events (MACEs).

**Results:** The training cohort consisted of 271 patients, while the testing cohort consisted of 119 patients. The nomogram considered diabetes, normalized low-frequency (nLF) power/normalized high-frequency (nHF) power, DC, cardiac troponin I (cTnI), post-PCI QFR of the target vessel. The model demonstrated excellent discriminative ability, with area under the curve (AUC) values of 0.874 (95% CI: 0.809-0.939) for 1-year MACE prediction in the training cohort and 0.893 (95% CI: 0.808-0.978) in the testing cohort. For 2-year MACE prediction, the AUC values were 0.882 (95% CI: 0.822-0.942) and 0.842 (95% CI: 0.724-0.960) in the training and testing cohorts.

**Conclusions:** We successfully developed and validated a risk stratification system that integrates baseline clinical characteristics (diabetes, cTnI levels), ANS parameters (nLF/nHF ratio, DC), and coronary physiological assessment (post-PCI QFR). This model effectively predicts MACEs in NSTE-ACS patients following PCI, providing valuable prognostic information for clinical decision-making.

## Introduction

In the contemporary era of interventional cardiology, significant advancements in primary percutaneous coronary intervention (PCI) have led to a substantial reduction in morbidity and mortality of patients with acute coronary syndrome (ACS), marking a pivotal milestone in cardiology^1^. However, despite achieving successful revascularization, clinical outcomes remain suboptimal, with over 20% of patients, particularly those with non-ST elevation ACS (NSTE-ACS), experiencing subsequent adverse coronary events^2^. This persistent clinical challenge underscores the critical need for early and precise risk stratification to optimize therapeutic strategies and implement tailored follow-up protocols, thereby improving long-term prognosis.

Several clinical risk scores for risk stratification of NSTE-ACS have been proposed, which are promoted by international guidelines for the management of patients with ACS^3^. Notably, the Thrombolysis in Myocardial Infarction (TIMI) and Global Registry of Acute Coronary Events (GRACE) risk scores have demonstrated robust predictive value in ACS prognosis. However, despite emerging evidence supporting the integration of novel cardiac biomarkers and physiological parameters into prognostic models, significant limitations persist in translating these risk assessment tools into practical applications for early postoperative and home-based rehabilitation strategies^4^. To address this critical gap, we developed an innovative risk prediction model specifically tailored for NSTE-ACS patients, enabling more precise individualized risk assessment and management.

Quantitative Flow Ratio (QFR), an innovative non-invasive fractional flow reserve assessment technology, utilizes coronary angiography imaging combined with three-dimensional vascular reconstruction and computational fluid dynamics analysis to quantify pressure gradients across coronary stenotic lesions, thereby enabling accurate diagnosis of myocardial ischemia^5^. Substantial clinical evidence has established a strong correlation between acute coronary syndrome (ACS) incidence and functionally significant coronary artery stenosis as determined by QFR, demonstrating its diagnostic utility in identifying ischemic pathologies in non-ST elevation ACS (NSTE-ACS) patients^6^. The autonomic nervous system (ANS) contributes to ACS progression through a complex mechanism involving local and systemic inflammation, creating a positive feedback loop that exacerbates atherosclerotic plaque formation and accelerates ACS development^7^. The integration of coronary physiological assessment with ANS evaluation has emerged as a promising approach to enhance prognostic precision in cardiovascular medicine^8^. Our preliminary investigations have revealed significant correlations between non-invasive ANS assessments and coronary physiology parameters measured by QFR^9^. Building upon these findings, we aimed to develop a predictive model that synergistically combines baseline clinical characteristics, QFR measurements, and ANS assessments to non-invasively predict cardiovascular outcomes in post-PCI NSTE-ACS patients.

## Methods

### Study population

Our study included consecutive patients diagnosed with NSTE-ACS according to international diagnostic criteria^10^, ^11^, encompassing both non-ST segment elevation myocardial infarction (NSTEMI) and unstable angina pectoris (UAP). Eligible participants underwent successful and complete percutaneous coronary intervention (PCI) with pre- and post-operative quantitative flow ratio (QFR) measurements at Renmin Hospital of Wuhan University between January 2018 and December 2020. To ensure a balanced distribution of outcome events, the study population was randomly allocated in a 7:3 ratio, yielding a training cohort of 271 patients and a testing cohort of 119 patients.

Exclusion criteria comprised: (1) cardiac conduction abnormalities (atrioventricular block, atrial fibrillation, or permanent pacemaker implantation); (2) acute ST-segment elevation myocardial infarction or chronic coronary syndrome; (3) active malignancies; (4) incomplete 24-hour Holter monitoring or QFR measurement data; (5) prior coronary artery bypass grafting; (6) angiographic evidence of prolonged coronary occlusions or left main coronary artery lesions; and (7) suboptimal angiographic image quality due to severe vessel overlap or excessive tortuosity of stenotic segments. All patients voluntarily provided informed consent by signing a consent form. This study adhered to the tenets of the Declaration of Helsinki and was conducted in accordance with the regulations of our medical center. The research protocol was reviewed and approved by the Ethics Committee of the Renmin Hospital of Wuhan University (No.WDRY2022-K257) and the study was registered on the China Clinical Trial Registry (No.ChiCTR2300068491).

### Blood sampling and laboratory analysis

Peripheral venous blood samples were collected from all participants prior to the procedure for comprehensive laboratory analysis. The biochemical profile included: (1) complete blood count parameters: white blood cell count (WBC), neutrophil count, lymphocyte count, platelet count (PLT), neutrophil-to-lymphocyte ratio (NLR), and platelet-to-lymphocyte ratio (PLR); (2) inflammatory markers: high-sensitivity C-reactive protein (hs-CRP); (3) cardiac biomarkers: cardiac troponin I (cTnI), creatine kinase-MB (CK-MB), and N-terminal pro-brain natriuretic peptide (NT-proBNP); and (4) lipid profile: low-density lipoprotein cholesterol (LDL-C), high-density lipoprotein cholesterol (HDL-C), triglycerides (TG), and total cholesterol (TC).

### Coronary angiography and QFR analysis

Coronary angiography was performed by certified interventional cardiologists following standardized protocols. The target vessel was identified as the coronary artery exhibiting the most severe stenosis in each case. Pre-procedural antiplatelet therapy, including loading doses of aspirin combined with either ticagrelor or clopidogrel, was administered in accordance with clinical guidelines^12^, ^13^. Percutaneous coronary intervention (PCI) was subsequently performed by experienced interventional cardiologists using second-generation drug-eluting stents, with procedural success defined as residual stenosis <20% in the target vessel accompanied by TIMI grade 3^13^. All PCI procedures were tailored to individual coronary anatomy and clinical presentations, performed by senior interventional cardiologists, followed by standardized post-procedural medical management. QFR analysis was conducted using the AngioPlus system (Pulse Medical Imaging Technology, Shanghai, China) following manufacturer-specified protocols^14^. In our study, three vessels QFR was defined as the sum of QFR in three vessels.

### Holter monitoring and heart rate variability analysis

All participants underwent continuous 24-hour 12-lead electrocardiographic monitoring following the procedure. The Holter monitoring data were analyzed to assess heart rate variability (HRV) parameters and 24-hour deceleration capacity (DC) 15. DC, an innovative non-invasive electrocardiographic metric, quantifies vagal nerve activity through the analysis of beat-to-beat interval oscillations, providing a reliable assessment of cardiac vagal tone^16^. HRV analysis was performed using both time-domain and frequency-domain methods. Time-domain parameters included: (1) standard deviation of normal-to-normal intervals (SDNN); (2) root mean square of successive differences (RMSSD); (3) standard deviation of 5-minute average NN intervals (SDANN); and (4) percentage of adjacent NN intervals differing by >50 ms (pNN50). The frequency-domain analysis comprised normalized low-frequency power (nLF) and normalized high-frequency power (nHF), with their ratio calculated as nLF/nHF. LF refers to the amplitude of the normal heartbeat intervals in the low-frequency range, and nLF refers to low-frequency power/(total power-very low frequency power)×100, which represents the sympathetic nerve activity. HF refers to the magnitude of the amplitude of the normal heartbeat intervals in the high-frequency range. nHF refers to high-frequency power/(total power-very low frequency power)×100, representing parasympathetic nerve activity. nLF/nHF represents of sympathetic-parasympathetic balance.

### Follow up

Clinical follow-up data were systematically collected through standardized telephone interviews and scheduled outpatient clinic visits following hospital discharge. The primary endpoint of this study was the occurrence of major adverse cardiovascular events (MACEs), defined as a composite of: (1) cardiac mortality; (2) unplanned revascularization; (3) recurrent acute myocardial infarction; and (4) hospital readmission due to UAP.

### Balance score development

The score derivation process involved sequential statistical analyses: initial univariate Cox regression identified potential predictors, followed by multivariate Cox regression analysis incorporating significant variables from the univariate analysis. Using the multivariate Cox regression results from the training cohort, we constructed predictive nomograms incorporating statistically significant prognostic factors. We developed a novel risk stratification tool, termed the Balance Score, which integrates baseline clinical parameters (diabetes status, cardiac troponin I [cTnI] levels, and quantitative flow ratio [QFR] measurements) with autonomic nervous system (ANS) indexes (nLF/nHF ratio and deceleration capacity [DC]) to non-invasively predict post-PCI cardiovascular events in non-ST elevation acute coronary syndrome (NSTE-ACS) patients. To enhance clinical utility, we developed an interactive web-based calculator for the Balance Score, enabling real-time, dynamic, and personalized risk assessment in clinical practice.

### Statistical analysis

All statistical analyses were performed using SPSS 23.0 (IBM Corporation, Chicago, IL, USA) and R version 3.6.1 (R Foundation for Statistical Computing, Vienna, Austria). Continuous variables were analyzed using parametric or non-parametric tests based on their distribution patterns: normally distributed data were expressed as mean ± standard deviation (SD) and compared using independent sample t-tests, while non-normally distributed data were presented as median (interquartile range, P25-P75) and analyzed using Mann-Whitney U tests. Categorical variables were expressed as frequencies (percentages) and compared using Fisher's exact test. The predictive performance of the model was evaluated through multiple approaches: (1) discrimination ability was assessed using receiver operating characteristic (ROC) curve analysis, with the area under the curve (AUC) and corresponding 95% confidence intervals (CI) calculated; (2) calibration was evaluated using calibration curves; (3) internal validation was performed through bootstrap resampling with 500 iterations to ensure model robustness; (4) clinical utility was determined using decision curve analysis (DCA). P-value <0.05 was considered statistically significant.

## Results

### Baseline characteristics and cohort distribution

The study enrolled 390 participants with a mean follow-up duration of 22.19 months, during which major adverse cardiovascular events (MACEs) occurred in 45 patients (11.5%). All study subjects were assigned random numbers and randomly divided into training cohort (n=271) and testing cohort (n=119) on the basis of 7:3 ratio. The mean follow-up durations were comparable between cohorts (training: 22.14 months; testing: 22.34 months), with MACE rates of 11.1% (n=30) and 12.6% (n=15) observed in the training and testing cohorts (**Table 1**). SDNN, rMSSD, Pnn50, nLF, nHF, nLF/nHF, average heart rate, NT-proBNP, cTnI, and number of diseased vessels, pre-PCI QFR of target vessel and total pre-PCI QFR were different between the training and testing cohorts (**Table 1**). The other baseline characteristics of the training and testing cohorts did not show significant differences (**Table 1**).

### Clinical outcomes

**Figure 1:**
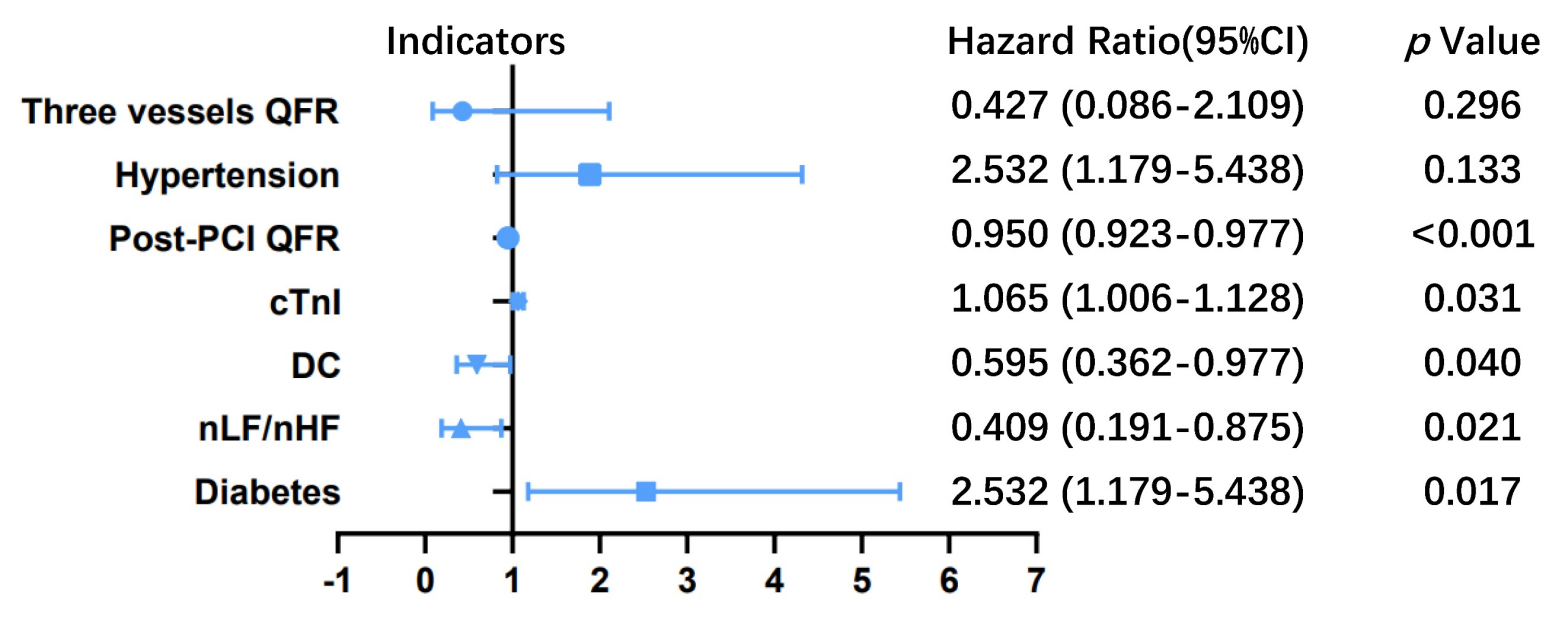
Hazard ratios for major adverse cardiovascular events (MACEs) in patients with non-ST-elevation acute coronary syndrome (NSTE-ACS) who underwent percutaneous coronary intervention (PCI). The blue line represents the 95% confidence intervals for hazard ratio, the blue dots represent the hazard ratio values of each variable, and there is a vertical line at x=1. The blue line does not intersect the dashed line representing that the 95% confidence intervals for the hazard ratio of the variables do not contain 1, the p-value was less than 0.05 and the variables were statistically significant for patient outcomes. nLF/nHF, ratio of normalized low-frequency to normalized high-frequency; DC, deceleration capacity; PCI, percutaneous coronary intervention; QFR, quantitative flow ratio; cTnI, cardiac troponin I.

In the training cohort, hypertension (P=0.133), diabetes (P=0.017), nLF/nHF (P=0.021), DC (P=0.040), cTnI (P=0.031), post-PCI QFR of target vessel (P<0.001) and the sum of QFR in three vessels (P<0.001) were potential correlated with increased risk of MACEs according to the result of univariate Cox regression analysis (**Table 2**). Multivariate Cox proportional hazards regression analysis revealed five independent predictors of MACEs: (1) diabetes (HR: 2.532; 95% CI: 1.179-5.438; P=0.017); (2) nLF/nHF ratio (HR: 0.409; 95% CI: 0.191-0.875; P=0.021); (3) cTnI level (HR: 1.065; 95% CI: 1.006-1.128; P=0.031); (4) post-PCI QFR of target vessel (HR: 0.950; 95% CI: 0.923-0.977; P<0.001); and (5) DC (HR: 0.409; 95% CI: 0.362-0.977; P=0.595) (**Table 2; Figure 1**).

### Construction of the nomogram in the training cohort

**Figure 2:**
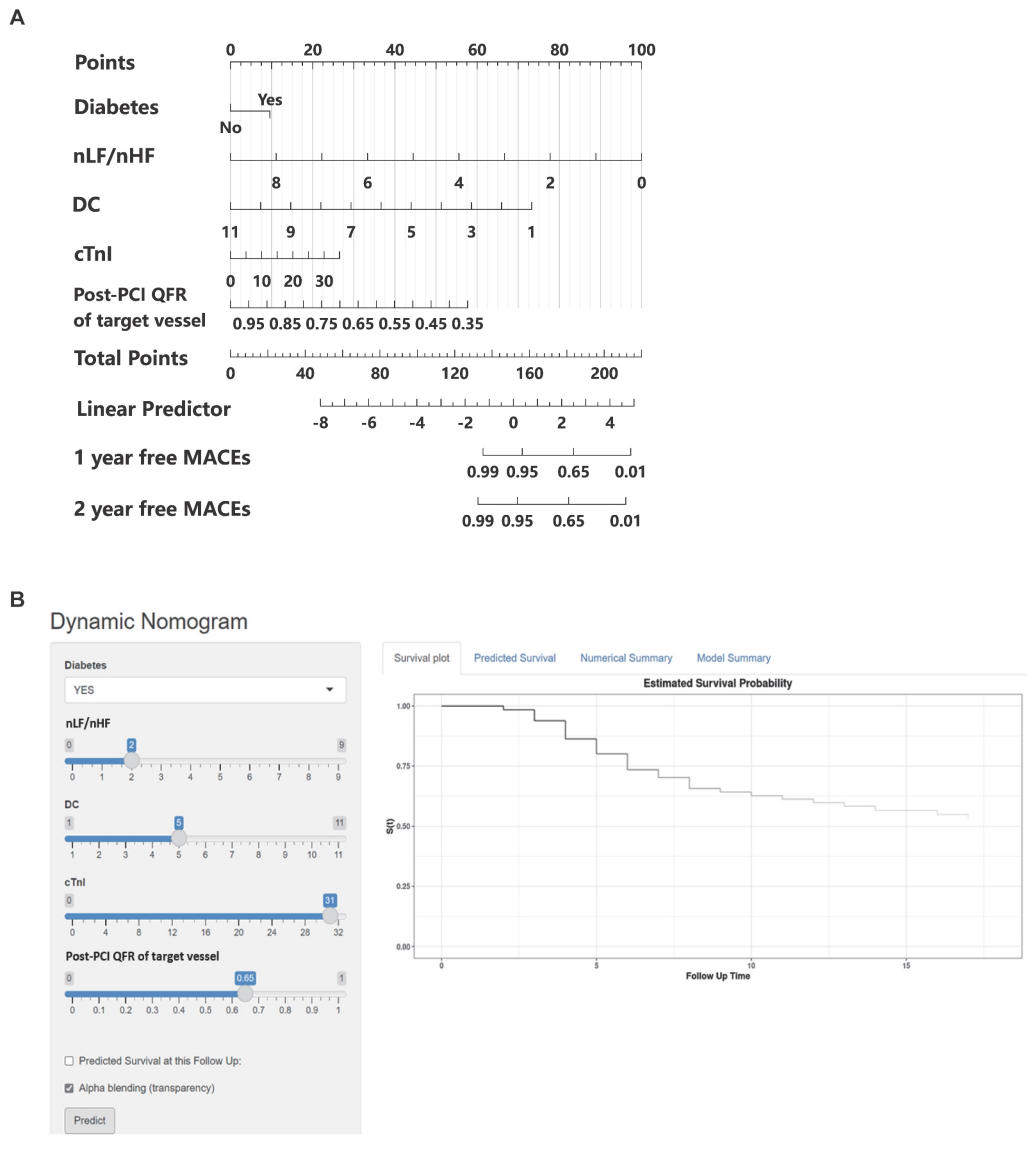
Nomogram for predicting the probability of MACEs in NSTE-ACS patients after PCI. (A) Each of the five clinical characteristics (diabetes, nLF/nHF, DC, cTnI, post-PCI QFR of target vessel) was assigned points by drawing a vertical line from its value to the top row. Number of points for each clinical characteristic is in the first line. The presence of characteristics is associated with a number of points generated employing the nomogram. The points for each characteristic are summed together to generate a total-points score. The total points correspond to the 1-year and 2-year probabilities of MACEs-free survival by drawing a vertical line to the bottom two rows. (B) A simple-to-use online dynamic nomogram for real-time calculation of Balance Score (https://nste-acs.shinyapps.io/BalanceS/). nLF/nHF, ratio of normalized low-frequency to normalized high-frequency; DC, deceleration capacity; QFR, quantitative flow ratio; cTnI, cardiac troponin I; MACEs, Major adverse cardiovascular events.

Based on the multivariable hazard ratios of identified predictors, we developed a nomogram to estimate the probability of 1- and 2-year major adverse cardiovascular event (MACE)-free survival in post-PCI non-ST elevation acute coronary syndrome (NSTE-ACS) patients (**Figure 2A**). This novel risk stratification tool termed the Balance Score, integrates significant prognostic factors into a user-friendly graphical representation. To facilitate clinical implementation, we created an interactive web-based nomogram (**Figure 2B**; https://nste-acs.shinyapps.io/BalanceS/). This platform, accessible via both mobile phones and computers, enables real-time risk calculation, allowing clinicians and patients to obtain instantaneous prognostic assessments regardless of time or location.

### Performance of the nomogram

**Figure 3:**
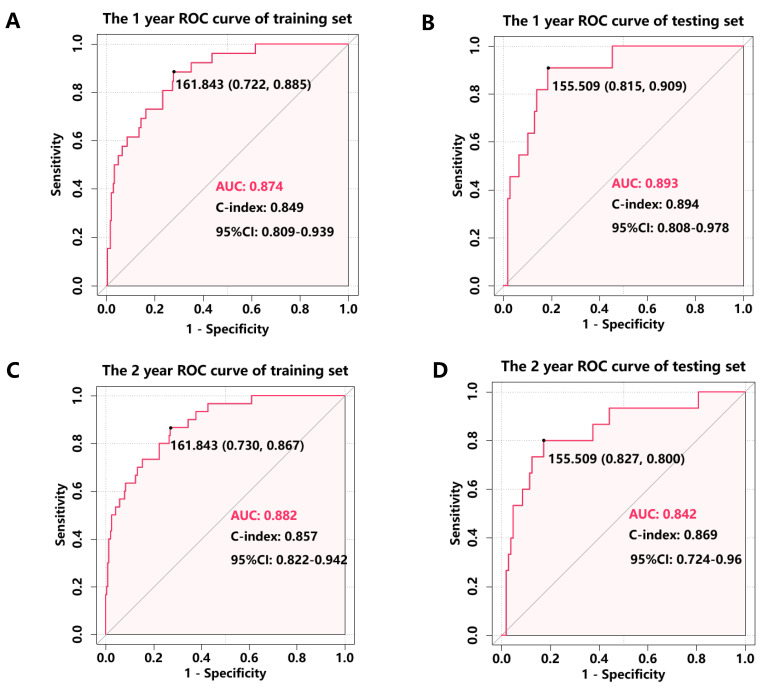
ROC analysis was performed to assess the accuracy of the nomogram for predicting 1- and 2- year MACEs-free survival in the training cohort and testing cohort. (A) 1-year ROC analysis of the accuracy of the nomogram in predicting MACEs-free survival in the training cohort; (B) 1-year ROC analysis of the accuracy of the nomogram in predicting MACEs-free survival in the testing cohort; (C) 2-year ROC analysis of the accuracy of the nomogram in predicting MACEs-free survival in the training cohort; (D) 2-year ROC analysis of the accuracy of the nomogram in predicting MACEs-free survival in the testing cohort.

**Figure 4:**
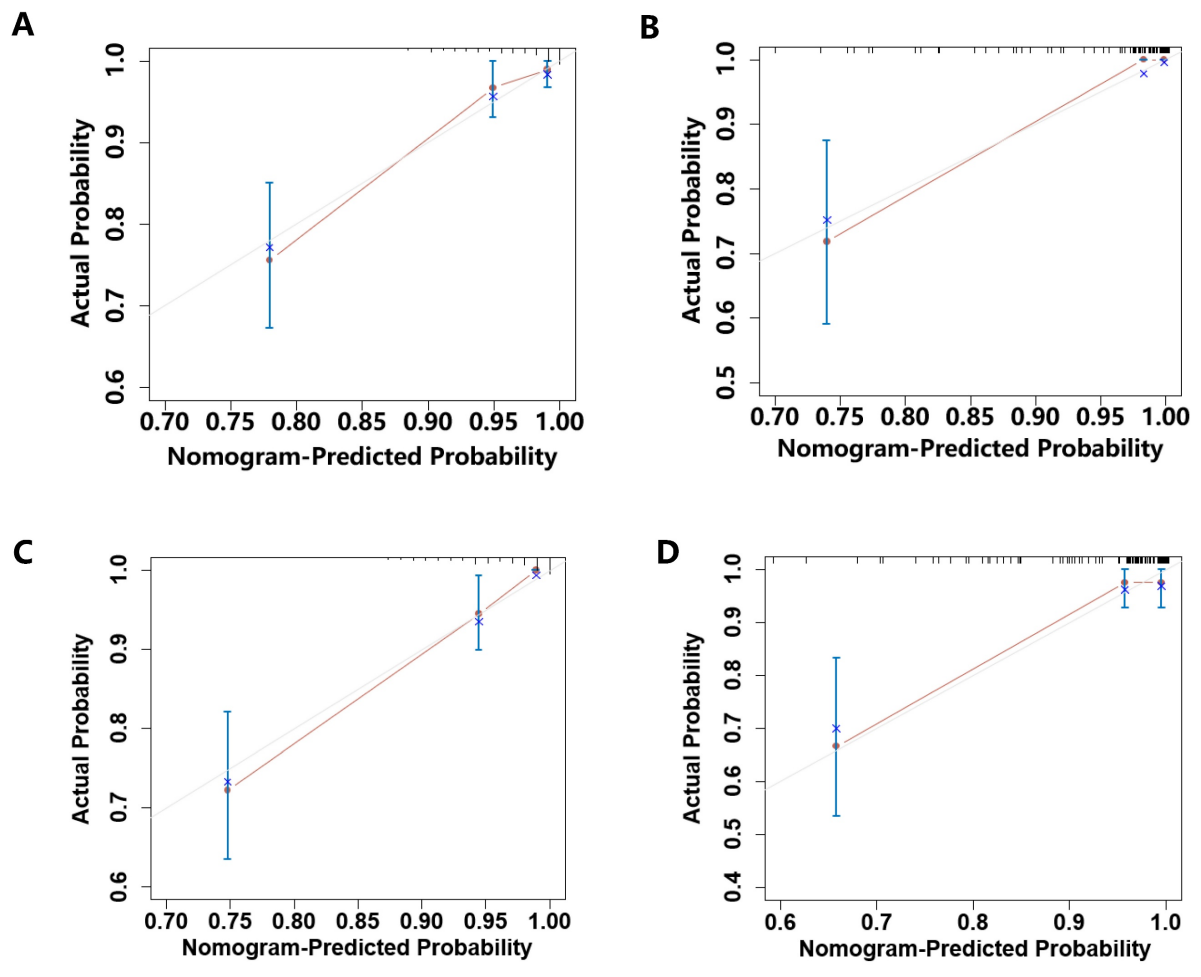
Calibration curves for the prediction of the risk for 1- and 2- year MACEs-free survival in the training cohort and testing cohort. (A)Calibration plot of the 1-year MACEs-free survival in the training cohort; (B)Calibration plot of the 1-year MACEs-free survival in the testing cohort; (C)Calibration plot of the 2-year MACEs-free survival in the training cohort; (D)Calibration plot of the 2-year MACEs-free survival in the testing cohort.

**Figure 5:**
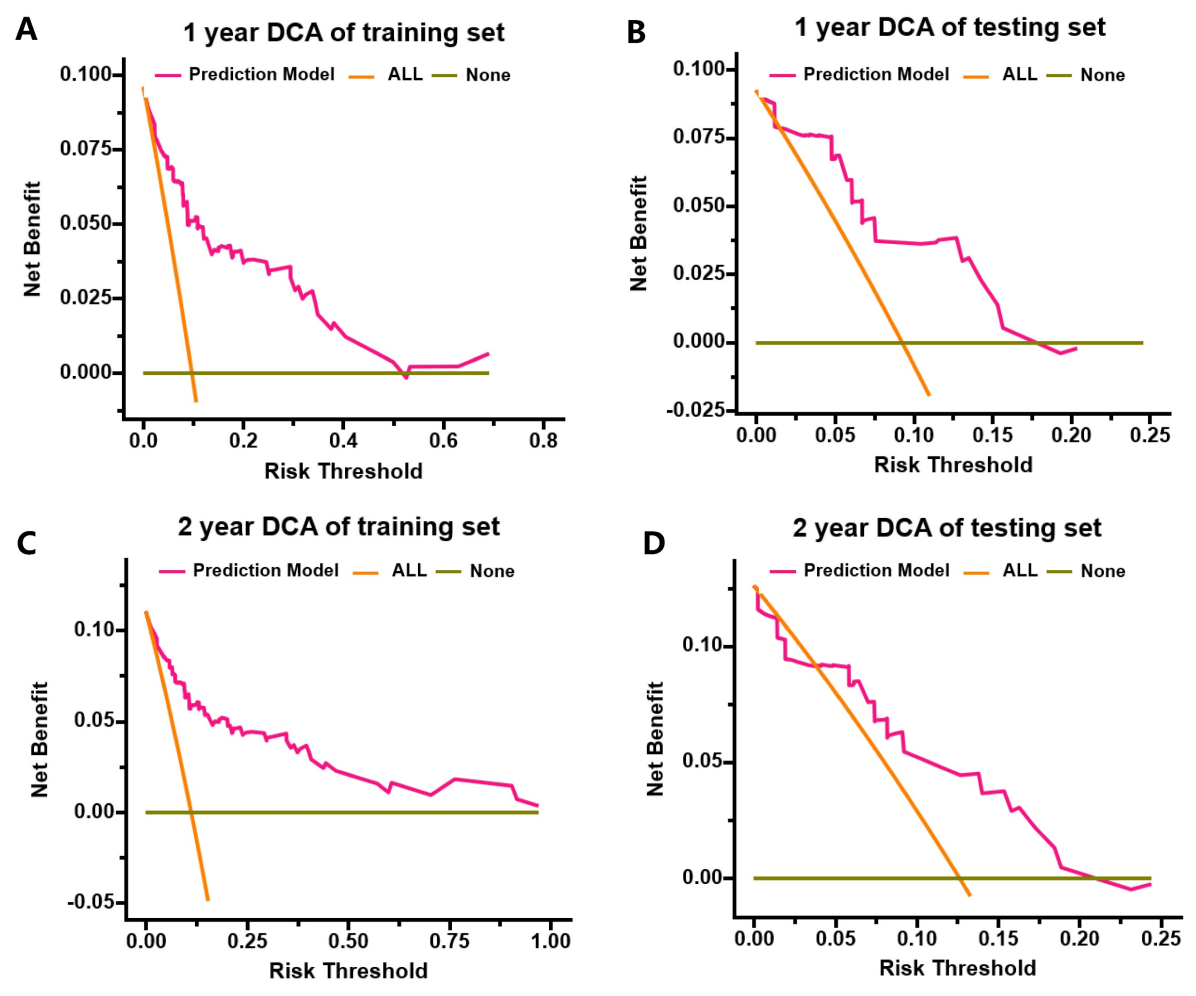
DCA for predicting 1- and 2- year MACEs-free survival in the training cohort and testing cohort. (A) DCA for predicting 1-year MACEs-free survival in the training cohort; (B) DCA for predicting 1-year MACEs-free survival in the testing cohort; (C) DCA for predicting 2-year MACEs-free survival in the training cohort; (D) DCA for predicting 2-year MACEs-free survival in the testing cohort.

The nomogram's discriminative ability, defined as its capacity to differentiate between patients with and without subsequent MACEs, was assessed using ROC curve analysis and Harrell's concordance index (C-index). In the training cohort, the nomogram demonstrated excellent discrimination, with AUC values of 0.874 (95% CI: 0.809-0.939) for 1-year MACE-free survival and 0.882 (95% CI: 0.822-0.942) for 2-year MACE-free survival (**Figure 3A, C**). Internal validation through bootstrapping with 500 resamples yielded bias-corrected C-index values of 0.849 and 0.857 for 1-year and 2-year predictions respectively. Analysis of the testing cohort demonstrated that the AUC was 0.893 (95% CI: 0.808-0.978) for the prediction of 1-year rates freedom from MACEs and 0.842 (95% CI: 0.724-0.96) for the prediction of 2-year rates freedom from MACEs (**Figure 3B, D**). Calibration plots revealed a close agreement between predicted and observed event probabilities in both training and testing cohorts (**Figure 4**), with bootstrapped validation (500 resamples) confirming the model's accuracy. To evaluate clinical utility, decision curve analysis (DCA) was performed across both cohorts (**Figure 5**). The results demonstrated the nomogram's robust clinical application value, supporting its implementation in routine clinical practice.

## Discussion

In the present study, we developed and validated a novel, user-friendly nomogram model, termed the Balance Score, to predict 1- and 2-year MACE-free survival in unselected NSTE-ACS patients. The model integrates multiple prognostic indicators, including baseline clinical characteristics (blood biomarkers [cardiac troponin I, cTnI]), coronary physiological parameters (post-PCI QFR of target vessels), and ANS assessments (nLF/nHF ratio and DC). According to the construction of the Balance Score, an easy-to-use online risk prediction webpage is generated simultaneously, which is convenient for dynamic, real-time and accurate calculation. The easy-to-use scoring system constructed in the present study can integrate and utilize existing clinical resources to make accurate evaluations without additional tests and does not impose additional time and financial burdens on physicians and patients. Furthermore, the simplicity and prognostic value of the Balance Score may be a helpful tool in future clinical practice.

Despite the widespread adoption of PCI as an effective treatment strategy for NSTE-ACS, a substantial proportion of patients remain at risk of mortality and adverse outcomes despite successful coronary revascularization^10^. This underscores the necessity for extending NSTE-ACS management beyond interventional therapy alone. Comprehensive risk factor management has emerged as a critical component in ACS prevention strategies. Emerging evidence highlights the intricate interplay between ANS dysfunction and systemic inflammation as fundamental pathophysiological mechanisms underlying ACS progression^17^, ^18^. Persistent inflammatory activation contributes to myocardial injury, cardiac dysfunction, and adverse ventricular remodelling, ultimately exacerbating cardiac performance^19^. Recent clinical studies have established residual inflammatory risk as an independent predictor of adverse cardiovascular events post-PCI^20^. Concurrently, ANS imbalance potentiates inflammatory responses, predisposing patients to reperfusion injury, malignant arrhythmias, and sudden cardiac death^21^, ^22^. These findings emphasize the importance of incorporating readily available clinical parameters, including ANS and inflammatory markers, into risk stratification models. The integration of diverse pathophysiological profiles significantly enhances risk prediction accuracy, particularly for post-PCI ACS patients^23^, ^24^, without imposing additional diagnostic burdens.

While the GRACE risk score demonstrates superior predictive accuracy for mortality and myocardial infarction compared to subjective physician assessment in ACS patients^10^, and the TIMI risk score provides a practical framework for early risk stratification^3^, these established models exhibit several limitations. Their predictive accuracy may be compromised by evolving disease patterns, advancements in healthcare quality, and changes in the natural history of ACS^10^. Recent studies have attempted to enhance risk prediction by integrating additional biomarkers, including postprandial glucose levels, cystatin C, interleukin-6, total bilirubin and DC with traditional risk scores^25^, ^26^. However, these enhanced models, while providing incremental prognostic value, may lack generalizability across diverse populations and healthcare systems^23^, ^27^. Notably, population-specific models, such as the 10-year atherosclerotic cardiovascular disease risk prediction model developed for the Chinese population, have shown promise in addressing these limitations^28^. Our previous studies have established the prognostic significance of ANS modulation in ACS, demonstrating its role in both the pathogenesis of NSTE-ACS and the reduction of reperfusion-related ventricular arrhythmias^29^. Building upon these findings, we identified a critical gap in current risk stratification approaches, the absence of an integrated model incorporating ANS coronary physiology, and clinical data for predicting MACEs in NSTE-ACS patients. To address this unmet need, we developed a novel, multiple-modality risk prediction model that synergistically combines readily available clinical parameters. This approach enables more precise identification of high-risk patients requiring intensive surveillance and preventive interventions while maintaining clinical feasibility and applicability.

QFR has emerged as a clinically valuable tool for assessing the functional severity of coronary artery stenosis. Substantial evidence demonstrates that QFR-derived coronary physiological assessment provides diagnostic accuracy comparable to fractional flow reserve (FFR), the current gold standard for evaluating functional coronary stenosis^14^. Our previous cohort study further established that integrating QFR-based coronary physiology with DC measurements offers incremental prognostic value beyond traditional cardiovascular risk factors for predicting MACEs in NSTE-ACS patients^9^. These findings align with existing literature supporting the prognostic utility of coronary physiological assessment in ACS. Diabetes mellitus demonstrates significant associations with adverse cardiovascular outcomes, including preclinical injury, coronary artery disease progression, and poor ACS prognosis^30^, ^31^. Furthermore, we corroborate previous evidence regarding the diagnostic and prognostic utility of cTnI as an early biomarker for AMI and its value in identifying high-risk CAD patients. It is worth noting that in our study, some indexes such as cTnI and nLF/nHF were statistically different between the training and testing groups. These two indexes were included as evaluation parameters for the Balance 2.0, this may raise doubts about the ability of the model to generalize among populations with different demographic characteristics. However, the statistical values of these two indexes are similar to their normal ranges in the clinic. Moreover, when adequate randomization is guaranteed, statistical differences cannot represent a balanced difference between the training group and the testing group^32^. The single-center design and limited sample size may affect the model's generalizability. Future validation in multicenter settings with diverse demographic characteristics is essential to fully evaluate the model's predictive performance and clinical applicability across different populations.

**Figure 6:**
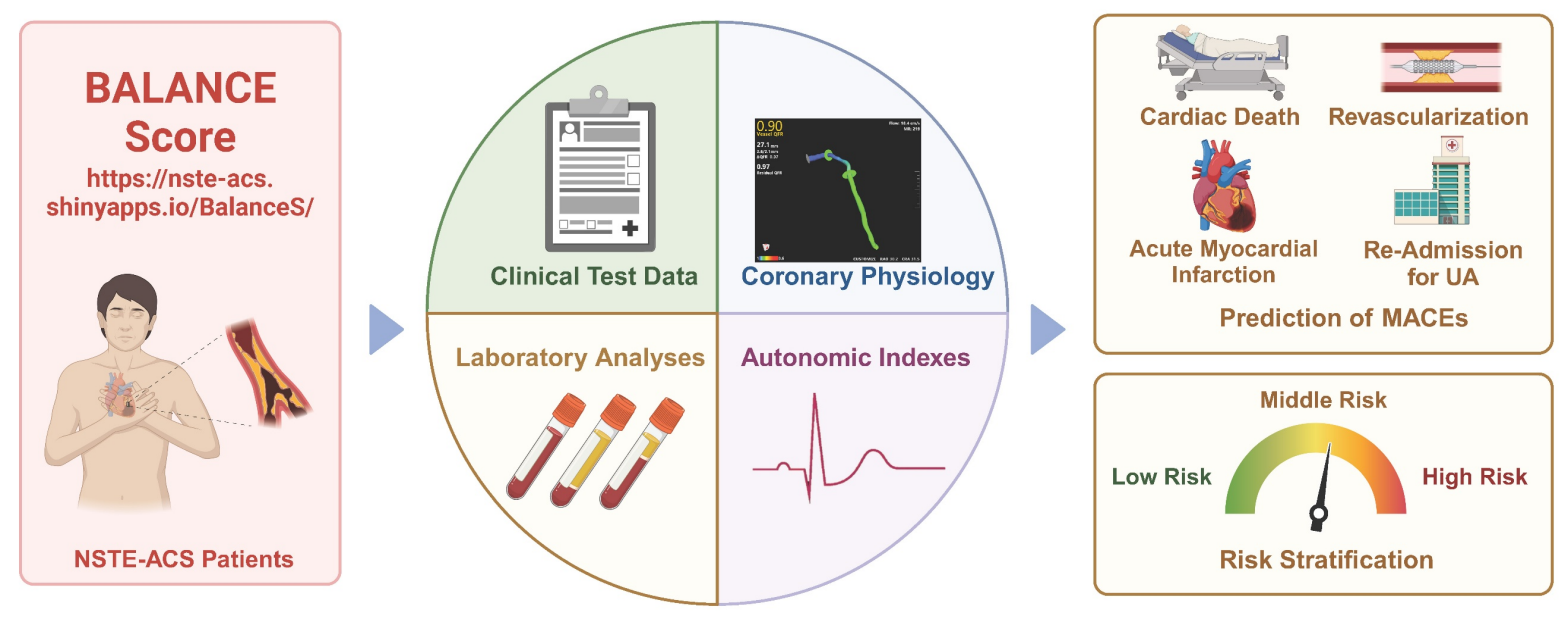
Central illustration. The integrated approach that incorporates multiple-modality data from baseline characteristics (Clinical text data, laboratory analyses and imaging data) and autonomic nervous system assessment were linked to risk stratification and non-invasively predict cardiovascular events. This model is easy-to-use and straightforward provides clinicians with a non-invasive and simple method for assessing the risk of MACEs in patients with NSTE-ACS.

As we enter the era of personalized and precision medicine, the characterizations of patients will directly or indirectly influence the therapeutic decisions of clinic practices. This paradigm shift coincides with the rapid adoption of data-driven approaches in modern healthcare, fueled by advancements in mobile health monitoring and digital technologies. The proliferation of wearable devices and smart home health technologies presents unprecedented opportunities for implementing visual, model-based personalized health management systems. In this study, we developed an innovative risk stratification model, termed the Balance Score, which integrates ANS evaluation, blood biomarkers, clinical data, and imaging parameters to predict MACEs in post-PCI NSTE-ACS patients. To enhance clinical utility, we implemented a user-friendly web-based platform that enables visual, personalized risk assessment through an intuitive interface requiring only five key clinical parameters. This integrated approach represents a significant advancement in patient-centered care, offering dynamic, real-time monitoring capabilities. The Balance Score system not only improves NSTE-ACS management but also contributes to the ongoing digital transformation of healthcare delivery, potentially serving as a model for future digital health initiatives (**Figure 6, central illustration**).

## Conclusion

Our novel nomogram-based prognostic model integrates multiple data modalities, including established cardiovascular risk factors (diabetes mellitus), laboratory biomarkers (cTnI), and QFR-derived imaging parameters. These parameters are readily accessible from routine clinical records and electronic health databases. The model further incorporates ANS indices (nLF/nHF ratio and DC), obtained through wearable devices, which serve as robust predictors of adverse cardiac events. This patient-centered approach represents a significant advancement in multidimensional, individualized risk assessment. By synergistically combining routinely available clinical data with advanced physiological parameters, our model not only enhances the management of NSTE-ACS patients but also contributes to the ongoing digital transformation of cardiovascular care delivery.

## Figures and Tables

**Table 1:** Baseline participant characteristics.

	Training set (n=271)	Testing set (n=119)	t/Z/χ2	P
Male n%	197 (72.7)	89 (74.8)	0.186	0.666
Age (years)	63.12±9.62	62.13±9.69	0.939	0.348
Hypertension Yes%	76 (28.0)	34 (28.6)	0.011	0.915
Diabetes Yes%	111 (41.0)	43 (36.1)	0.806	0.369
Current smoker Yes%	119 (43.9)	55 (46.2)	0.178	0.673
Current drinker Yes%	60 (22.1)	35 (29.4)	2.373	0.123
Family history of CAD Yes%	34 (12.5)	22 (18.5)	2.374	0.123
Previous PCI Yes%	74 (27.3)	34 (28.6)	0.066	0.797
Previous myocardial infarction Yes%	22 (8.1)	13 (10.9)	0.797	0.372
WBC (×10^9^/L)	6.63±1.93	6.59±1.90	0.181	0.856
Neutrophils (×10^9^/L)	4.04±1.33	4.15±1.48	0.731	0.465
Lymphocytes (×10^9^/L)	1.72±0.61	1.66±0.60	0.973	0.331
NLR (ratio)	2.38 (1.81,3.08)	2.48 (1.92,3.21)	0.976	0.329
PLTs (×10^9^/L)	209.83±62.01	197.59±52.93	1.874	0.062
PLR(ratio)	134.46±58.22	130.67±45.22	0.631	0.529
TG (mmol/L)	1.48 (1.07,2.22)	1.59 (1.15,2.62)	1.291	0.197
TC (mmol/L)	4.07±1.29	3.97±1.37	0.651	0.515
HDL-C (mmol/L)	1.05±0.28	1.06±0.32	0.368	0.713
LDL-C (mmol/L)	2.35±0.98	2.43±1.01	0.722	0.470
Pro-NT BNP (ng/L)	217.70 (86.09,607.60)	145.60 (62.69,584.61)	2.135	0.033
Average heart rate (bpm)	70.68±7.63	73.11±10.67	2.249	0.026
SDNN (ms)	119.69±47.63	109.13±31.81	2.570	0.011
SDANN (ms)	94.67±34.34	89.87±25.66	1.527	0.128
rMSSD (ms)	40.00 (27.00,61.00)	34.00 (22.00,52.00)	2.790	0.005
Pnn50	5.00 (20.0,14.00)	3.00 (1.00,8.00)	3.336	0.001
Normalized low-frequency	54.95±14.82	67.55±13.58	8.200	<0.001
Normalized high-frequency	45.05±14.82	32.45±13.58	8.200	<0.001
nLF/nHF	1.30 (0.75,1.91)	2.15 (1.40,3.85)	7.308	<0.001
DC (ms)	3.43±1.54	3.75±1.35	1.911	0.057
CK-MB (U/L)	1.47 (0.94,2.93)	1.28 (0.70,2.47)	1.642	0.101
Cardiac troponin I (ng/mL)	0.04 (0.01,1.08)	0.01 (0.01,0.07)	4.156	<0.001
Number of diseased vessels	2.09±0.82	1.80±0.71	3.546	<0.001
Target vessel			5.840	0.054
LAD	128 (47.2)	72 (60.5)		
LCX	56 (20.7)	18 (15.1)		
RCA	87 (32.1)	29 (24.4)		
Number of stents	1.76±0.99	1.65±0.86	0.887	0.376
Pre-PCI QFR of target vessel	0.64±0.15	0.52±0.24	5.128	<0.001
Post-PCI QFR of target vessel	0.90±0.10	0.91±0.06	0.287	0.775
The sum of Pre-PCI QFR in three vessels	2.37±0.32	2.22±0.40	3.497	0.001
The sum of Post-PCI QFR in three vessels	2.70±0.23	2.71±0.23	0.224	0.823
MACEs	30 (11.1)	15 (12.6)	0.191	0.662
Cardiac death	6 (2.2)	4 (3.4)	0.436	0.509
Revascularization	14 (5.2)	5 (4.2)	0.166	0.684
AMI	4 (1.5)	3 (2.5)	0.091	0.763
Re-admission for unstable angina	9 (3.3)	4 (3.4)	0.000	0.984

CAD, coronary artery disease; ACS, acute coronary syndrome; WBC, white blood cell; TG, triglyceride; TC, total cholesterol; NLR, neutrophil to lymphocyte ratio; PLT, platelet counts; PLR, platelet-to-lymphocyte ratio; HDL-C, high-density lipoprotein; LDL-C, low density lipoprotein; nLF/nHF, ratio of normalized low-frequency to normalized high-frequency; LAD; left anterior descending coronary artery; LCX; left circumflex; RCA; right coronary artery; PCI, percutaneous coronary intervention; QFR, quantitative flow ratio; AMI, acute myocardial infarction.

**Table 2:** Univariate and multivariable Cox regression analysis of factors associated with MACEs in the training cohort.

Variable	Univariate			Multivariate		
	HR	95% CI	P	HR	95% CI	P
Male n%	0.996	0.444-2.238	0.993			
Age	0.984	0.949-1.020	0.385			
Hypertension Yes%	2.349	1.147-4.814	0.020	1.886	0.824-4.316	0.133
Diabetes Yes%	2.238	1.078-4.646	0.031	2.532	1.179-5.438	0.017
Current smoker Yes%	1.287	0.629-2.633	0.490			
Current drinker Yes%	1.071	0.460-2.497	0.873			
Family history of CAD Yes%	0.760	0.231-2.505	0.652			
Previous PCI Yes%	1.584	0.754-3.329	0.225			
Previous myocardial infarction Yes%	1.250	0.379-4.119	0.714			
WBC (×10^9^/L)	1.045	0.875-1.248	0.626			
Neutrophils (×109/L)	1.072	0.825-1.394	0.601			
Lymphocytes (×109/L)	1.071	0.611-1.878	0.810			
NLR	0.952	0.692-1.311	0.763			
PLTs (×109/L)	0.999	0.993-1.005	0.748			
PLR	0.996	0.989-1.004	0.321			
TG mmol/L	0.754	0.509-1.118	0.160			
TC mmol/L	1.057	0.820-1.362	0.671			
HDL-C mmol/L	2.060	0.658-6.450	0.214			
LDL-C mmol/L	0.964	0.664-1.401	0.848			
Pro-NT BNP ng/L	0.999	0.999-1.001	0.331			
Average heart rate bpm	1.027	0.984-1.072	0.221			
SDNN ms	1.003	0.996-1.009	0.420			
SDANN ms	1.006	0.997-1.015	0.197			
rMSSD ms	1.001	0.993-1.009	0.878			
Pnn50	1.002	0.979-1.025	0.859			
Normalized low-frequency	0.962	0.938-0.986	0.002			
Normalized high-frequency	1.040	1.014-1.066	0.002			
nLF/nHF	0.418	0.226-0.772	0.005	0.409	0.191-0.875	0.021
DC ms	0.464	0.279-0.772	0.003	0.595	0.362-0.977	0.040
CK-MB U/L	1.008	0.996-1.020	0.206			
Cardiac troponin I	1.060	1.005-1.117	0.031	1.065	1.006-1.128	0.031
Number of diseased vessels	1.019	0.656-1.584	0.932			
Target vessel						
LCX vs LAD	1.311	0.566-3.034	0.527			
RCA vs LAD	0.429	0.175-1.050	0.064			
Number of stents	1.033	0.732-1.458	0.852			
Pre-PCI QFR of target vessel	0.537	0.060-4.783	0.577			
Post-PCI QFR of target vessel	0.900	0.848-0.955	<0.001	0.950	0.923-0.977	<0.001
The sum of Pre-PCI QFR in three vessels	0.468	0.181-1.209	0.117			
The sum of Post-PCI QFR in three vessels	0.125	0.046-0.336	<0.001	0.427	0.086-2.109	0.296

CAD, coronary artery disease; ACS, acute coronary syndrome; WBC, white blood cell; TG, triglyceride; TC, total cholesterol; NLR, neutrophil to lymphocyte ratio; PLT, platelet counts; PLR, platelet-to-lymphocyte ratio; HDL-C, high-density lipoprotein; LDL-C, low density lipoprotein; nLF/nHF, ratio of normalized low-frequency to normalized high-frequency; LAD; left anterior descending coronary artery; LCX; left circumflex; RCA; right coronary artery; PCI, percutaneous coronary intervention; QFR, quantitative flow ratio; AMI, acute myocardial infarction; HR, hazard ratio; CI, confidence interval.

## Abbreviations

ANS: autonomic nervous system
QFR: quantitative flow ratio
ACS: acute coronary syndrome
NSTE-ACS: non-ST-elevation ACS
PCI: percutaneous coronary intervention
DC: deceleration capacity
HRV: heart rate variability
MACEs: major adverse cardiac events
nLF: normalized low-frequency
nHF: normalized high-frequency
cTnI: cardiac troponin I
AUC: area under the curve
NSTE-ACS: Non-ST-elevation ACS
ANS: Autonomic nervous system
QFR: Quantitative flow ratio
MACEs: Major adverse cardiac events
